# Synergistically enhanced selective intracellular uptake of anticancer drug carrier comprising folic acid-conjugated hydrogels containing magnetite nanoparticles

**DOI:** 10.1038/srep41090

**Published:** 2017-01-20

**Authors:** Haneul Kim, Ara Jo, Seulgi Baek, Daeun Lim, Soon-Yong Park, Soo Kyung Cho, Jin Woong Chung, Jinhwan Yoon

**Affiliations:** 1 Department of Chemistry, Dong-A University, 37 Nakdong-daero 550 beon-gil, Saha-gu, Busan, 49315, Korea; 2 Department of Biological Science, Dong-A University, 37 Nakdong-daero 550 beon-gil, Saha-gu, Busan, 49315, Korea

## Abstract

Targeted drug delivery has long been extensively researched since drug delivery and release at the diseased site with minimum dosage realizes the effective therapy without adverse side effects. In this work, to achieve enhanced intracellular uptake of anticancer drug carriers for efficient chemo-therapy, we have designed targeted multifunctional anticancer drug carrier hydrogels. Temperature-responsive poly(*N*-isopropylacrylamide) (PNIPAm) hydrogel core containing superparamagnetic magnetite nanoparticles (MNP) were prepared using precipitation polymerization, and further polymerized with amine-functionalized copolymer shell to facilitate the conjugation of targeting ligand. Then, folic acid, specific targeting ligand for cervical cancer cell line (HeLa), was conjugated on the hydrogel surface, yielding the ligand conjugated hybrid hydrogels. We revealed that enhanced intracellular uptake by HeLa cells *in vitro* was enabled by both magnetic attraction and receptor-mediated endocytosis, which were contributed by MNP and folic acid, respectively. Furthermore, site-specific uptake of the developed carrier was confirmed by incubating with several other cell lines. Based on synergistically enhanced intracellular uptake, efficient cytotoxicity and apoptotic activity of HeLa cells incubated with anticancer drug loaded hybrid hydrogels were successfully achieved. The developed dual-targeted hybrid hydrogels are expected to provide a platform for the next generation intelligent drug delivery systems.

Cancer is one of the leading causes of death worldwide despite the recent advances in cancer therapeutics. Although many cancers can be effectively treated by simple surgery at the early stage, prolonged and intensive chemotherapy or radiotherapy are often required for the treatment of more advanced or metastatic cancers, which may accompany various side effects such as drug resistance and toxicity against normal cells surrounding the tumors^1^. Various drug carriers have been developed to overcome these problems^2^,^3^,^4^, however, there are still several concerns limiting clinical applications, such as drug carrier cytotoxicity, low permeability, and reduced ability to adjust the drug-dosages. Thus, development of more efficacious system is required for reduced systemic toxicity and increased efficiency.

Hydrogels are being intensively researched in biomedical fields as a key component for drug delivery^4^, due to their high water content, biocompatibility, and elastic property. They can provide biocompatible wet scaffolds for cells, tissues and drugs, which is very similar to natural environments. Especially, hydrogel beads are advantageous in drug delivery applications due to the fact that almost any material suspended in water can be loaded into the hydrogel matrix and the release can easily be manipulated through the choice of network materials and crosslinking density^5^. Furthermore, they can undergo the volume phase transition in response to the external-stimuli such as temperature, pH, solvent polarity, and light, enabling on-demand release of the loaded materials. For example, crosslinked poly(*N*-isopropylacrylamide) (PNIPAm) is a well-documented temperature-responsive hydrogel that show reversible lower critical solution temperature (LCST) phase transition around the temperature of human body^6^. Drug in solution can be loaded into the swollen state at lower temperature and release can be achieved at body temperature when the liquid contents are squeezed out through LCST behavior.

As well as the external stimuli-triggered drug release, a targeted delivery is also highly desirable for the intelligent drug carriers, since the concentration of the medication can be increased only in necessary parts of the body^7^. Smart delivery at the right point with minimum dosage administration allow the effective drug therapy without adverse side effects. Although drug carriers can be accumulated at the tumor site by enhanced permeability and retention (EPR) effect of leaky neoplastic vasculature around the tumor tissue^8^, advanced active targeting technology is greatly desired for the effective delivery.

To achieve the active targeting of the hydrogel carrier, much research efforts such as conjugation of targeting ligands or incorporation of magnetic nanoparticles (MNPs) have been performed. Conjugation of specific affinity ligand including antibody^9^, peptide^10^, aptamer^11^, glycosaminoglycan^12^ and vitamin^13^ provides selective delivery of drugs to target cells via receptor-mediated endocytosis. Hydrogels containing MNP can be used in the magnet-guided drug delivery^14^. Use of magnetic field to increase magnetic nanoparticle accumulation in target site has been realized both *in vitro*^15^ and *in vivo*^16^. However, this approach alone does not ensure the specific interaction of drug carriers with target cells. Therefore, dual-targeting system which can be achieved by combining ligand conjugation and magnet guiding would synergistically enhance the therapeutic efficiency of hydrogel vehicles.

In this study, to render the dual-targeted stimuli-responsive drug carriers, we pursued to prepare ligand conjugated hydrogels containing MNPs for the delivery of anticancer drug. Firstly, MNP incorporated PNIPAm-based core was prepared by precipitation polymerization, then its surface was further modified with amine functional group by subsequent polymerization of NIPAm and allylamine (AA). Secondly, folic acid (FA) was conjugated on the surface of hydrogel via EDC/NHS coupling, yielding the dual-targeted stimuli-responsive drug carrier hydrogel. Physicochemical properties of the generated hydrogels were characterized by dynamic light scattering (DLS), scanning electron microscopy (SEM), transmission electron microscopy (TEM), and UV/Vis spectroscopy. In order to study the potential for guided and sustained release, magnet response and drug release profile were also studied. Influence of the targeting ligand conjugation and the presence of magnet on the intracellular uptake and distribution were observed using confocal laser scanning microscopy (CLSM) with several cell lines. Finally, efficiency of anticancer drug-loaded MHG-FA were measured using MTT assay and apoptotic activity assay.

## Results and Discussion

### Hydrogel design and synthesis

**Figure 1:**
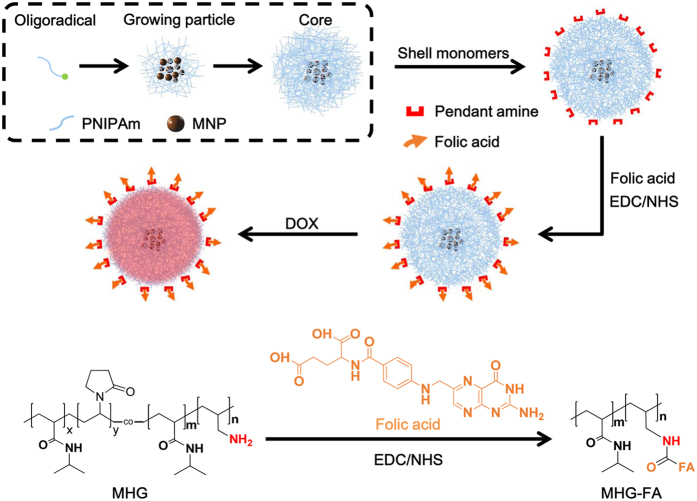
Schematic illustration of MHG-FA hydrogel synthesis. Hydrogel core containing MNPs and amine-functionalized shell were prepared by precipitation polymerization. Targeting ligand, folic acid (FA), was conjugated via EDC/NHS chemistry. Anticancer drug, DOX, was loaded at 4 °C.

Figure 1 shows the synthetic scheme for the preparation of double targeted stimuli-responsive drug-carriers. MHG composed of the hydrogel core containing MNPs and amine-functionalized shell was prepared by precipitation polymerization method. Briefly, p(NIPAm-co-VP) hydrogel core containing MNPs was prepared first, followed by p(NIPAm-co-AA) shell formation. The deswollen state of core particle at 70 °C minimized the penetration of shell mixtures during the shell formation, which enabled free amine moieties to be exposed on the surface. Targeting ligand FA was conjugated to the free amine on the shell surface via EDC/NHS chemistry. Finally, anticancer drug DOX was loaded at 4 °C.

Precipitation polymerization used in this work provides several advantages that (1) relatively uniform hydrogels can be obtained without the use of any surfactant or stabilizer; (2) hydrogel size can be controlled from several tens of nanometer to several micrometers by adjusting the concentration of the monomer or the stirring rate; and (3) composition of the hydrogel can easily be manipulated by simple addition during the polymerization. These features enable simple and easy component variation of the core (NIPAm, VP, and bisAA) and the shell (NIPAm, AA and bisAA). PNIPAm is a well-known temperature responsive polymer which exhibits reversible volume change near LCST of 32 °C^6^. To apply the drug carriers *in vivo*, LCST above the body temperature is preferred. Thus, the hydrogel was designed to induce sharp volume phase transition slightly above the body temperature by copolymerization of VP with NIPAm. In our previous study, we revealed that the incorporating hydrophilic VP moieties into the PNIPAm chain lead to the increase of LCST^17^. For the shell formation, AA was added as amine source for conjugating FA through amide coupling and bisAA was used as the crosslinker.

**Figure 2:**
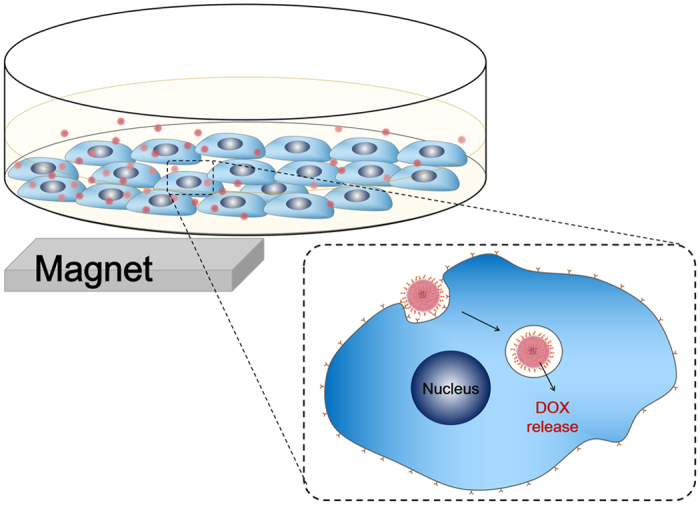
Schematic illustration of dual-targeted uptake of hydrogels and intracellular drug release. Hydrogels in circulation accumulate in the target tissue by the combination of EPR effect of the leaky vasculature and strong local magnetic field applied by the external magnet. Then, uptake of FA-conjugated hydrogels into cells is mediated by the folate receptor via receptor-mediated endocytosis. Image courtesy by Ara Jo.

Active dual-targeting is achieved by MNP in the core and FA conjugated on the hydrogel surface. As illustrated in Fig. 2, superparamagnetic MNP can be attracted to external magnet and therefore enhanced accumulation in the target site can be achieved. EPR effect of the leaky vasculature at tumor site is a well-documented effect^8^. This in combination with strong local magnetic field can guide hydrogel containing MNP to increase accumulation in the target tissue (preferably tumor site). The idea of using magnetic micro- and nanoparticles for *in vitro* biomedical applications such as cell sorting and immunoassay has long been widely utilized^18^,^19^,^20^. Recently, Wang *et al*. demonstrated enhanced cellular uptake of iron oxide-polypyrrole-PEG nanoparticles *in vitro* driven by the presence of magnet for anticancer drug delivery^15^. Moreover, MNP can further provide possibility as T2 contrast agent in magnetic resonance imaging^21^. Since MNP can generate heat under light irradiation, they can also be used in the hyperthermia treatment of tumors or triggering the light-induced volume phase transition of the temperature-responsive hydrogels^22^.

FA was selected as the targeting ligand since it is one of the best-characterized ligands to be exploited for targeting cancer cells^23^. Uptake of FA-conjugated particles into cells is mediated by the folate receptor via receptor-mediated endocytosis (Fig. 2). Since FA conjugation is achieved by EDC/NHS reaction through the amide bond formation between amino group of hydrogel and the carboxyl group of FA, FA can easily be substituted by other targeting ligands of choice. Enhanced accumulation at tumor site in combination with targeting ligand can synergistically increase the uptake of the hydrogel by cancer cells and therefore increased therapeutic effect can be achieved.

### Characterization

The size and surface charge of the core, core-shell hydrogel (MHG), and folic acid-conjugated core-shell hydrogel (MHG-FA) were determined by DLS. As summarized in Table 1, hydrodynamic sizes increased from 160.2 to 191.2 and 219.5 while PDI remained relatively low (0.111, 0.157, and 0.125, respectively). These results mean that the size of the hydrogel increase with the formation of the shell and conjugation of bulk targeting ligand.

**Figure 3:**
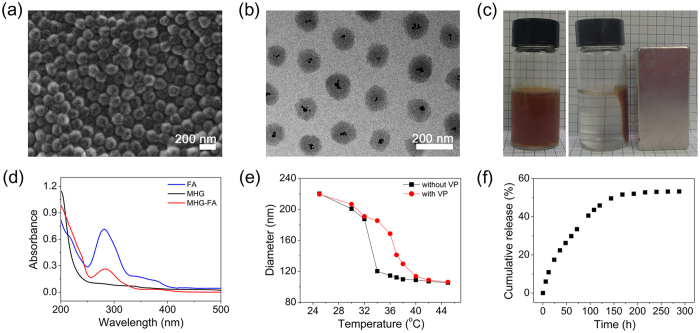
Characterization of hydrogels. Morphologies of hydrogels observed by (**a**) SEM and (**b**) TEM. (**c**) Photos of hydrogel solutions in the absence(left) and presence of a magnet (right). (**c**) UV-vis absorbance spectra of FA (blue line), MHG (black line) and MHG-FA (red line). (**e**) Temperature-dependent size change of PNIPAm hydrogels copolymerized without (black line) and with VP (red line). (**f**) DOX release from hydrogels over time at 37 °C.

The morphology of the samples was observed by SEM (Fig. 3a) and TEM (Fig. 3b). Hydrogels were relatively uniform in size and spherical in shape with diameter slightly less than 200 nm. Smaller particle size observed in dried samples of SEM and TEM than hydrated hydrogels in DLS measurement is a well-known phenomenon^24^,^25^. As shown in Fig. 3b, TEM image reveals the presence of MNP as black dots mainly in the core of MHGs, indicating that polymerization at the deswollen state of core at 70 °C both minimized the interpenetration of shell components into the core and prevented the MNPs from popping out during the synthesis. To confirm whether magnet-guided targeting can be achieved, MHGs suspended in distilled water were exposed to a magnet. As shown in Fig. 3c, in less than 10 min, clear separation was achieved indicating strong attraction to a local magnetic field.

Zeta potential values of core, MHG and MHG-FA were −9 mV, +10.2 mV and −20.6 mV, respectively (Table 1). This indirectly confirmed shell formation as positive surface charge indicated the presence of amine group on the shell surface and FA conjugation as shift back to negative surface charge indicated the presence of free carboxyl group remaining in the FA. Figure 3d show the UV/Vis spectra for the free FA, MHG, and MHG-FA. While MHG (black line) did not show any absorption peak, FA (blue line) showed strong absorption at ~260 nm due to π-π* transitions. MHG-FA (red line) showed absorption peak at ~280 nm, confirming that the conjugation of FA on the MHG. The slight shift from ~260 nm to ~280 nm may be attributed from the formation of amide bonding between carboxyl group of FA and amino group of hydrogel.

DOX, model anticancer drug used in this study, is an antineoplastic agent commonly used to treat tumors^26^. Drug loading was carried out at 4 °C where PNIPAm hydrogel is fully swollen and thus more drugs can be incorporated into the expanded network. The loading efficiency measured by weight was 27.5, 31.1, and 34.1%, slightly increasing with higher DOX concentration (0.26 mg, 0.52 mg, and 1.3 mg, respectively, in 3 mL PBS). Incorporation of 12.5 mol % VP increased the LCST from ~32 °C to ~37 °C (Fig. 3e). The hydrophilic functional group of VP promotes the hydration of polymer backbone, retarding polymer aggregation and hence leading an upward LCST shift. As hypothesized, this greatly impacted the drug release profile. 12.5 mol % of VP incorporation achieved sustained DOX release over ~100 h (Fig. 3f) while PNIPAm alone released DOX in just over 6 h (Figure S1 in Supplementary information). Burst release of anticancer drug in few hours is a major problem in drug delivery as it greatly increases the chance of side effect^27^.

### Dual-targeted cellular uptake and distribution

Intracellular uptake and distribution of hydrogels were observed using CLSM. Three different cell lines were tested including folate-receptor (FR)-positive HeLa and FR-negative MDA and Nuff cells^28^,^29^. In order to test the magnet-guided uptake of MHGs, W_magnet group culture plates were placed on magnet and incubated with the hydrogels for 24 h. MHGs appear as green dots from copolymerized fluorescein-o-methacrylate (FMA), a green fluorescent monomer. Nucleus were stained blue with Hoechst 33342 and plasma membranes were stained orange to better distinguish endocytosed hydrogels from those outside the cellular membrane. Z-stack images were also taken to confirm that hydrogels were inside the cell not adhered on the cell surface (Figures S2 and S3).

**Figure 4:**
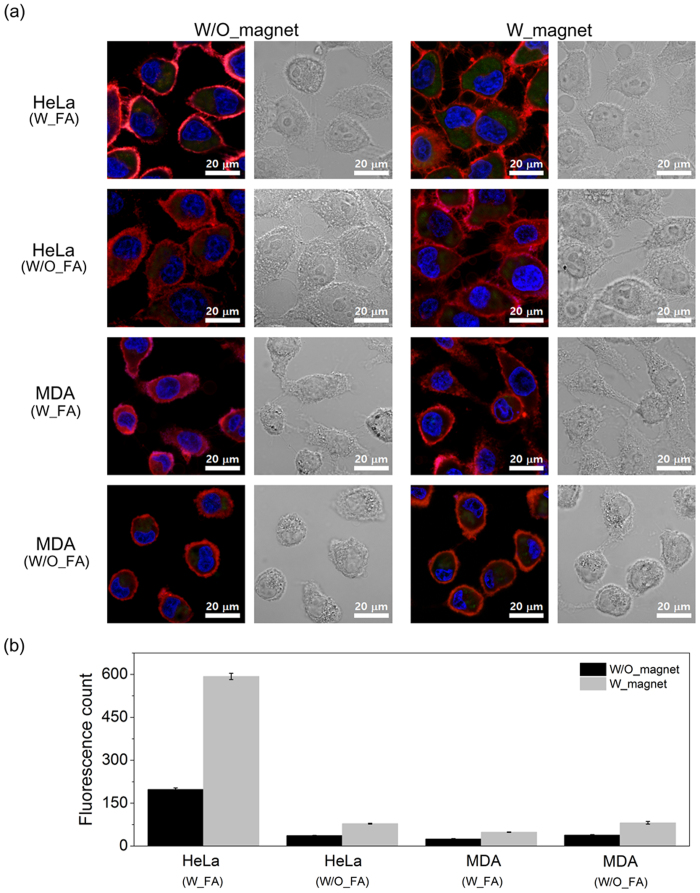
Intracellular localization and cellular uptake of hydrogels by HeLa and MDA-MB-231 cells. (**a**) Intracellular localization of hydrogels in HeLa and MDA-MB-231 cells recorded using CLSM. Cells were incubated with 100 μg/mL of MHG-FA (W_FA) or MHG (W/O_FA) without magnet (W/O_magnet, images on the left) or with magnet (W_magnet, images on the right). MHGs appear as green dots from copolymerized FMA, a green fluorescent monomer. Nuclei were labeled blue with Hoechst 33342 and cell plasma membranes were stained orange. (**b**) Quantitative cellular uptake of hydrogels by HeLa and MDA-MB-231 cells counted using Image J. Error bars represent standard deviation of three representative cells.

FA conjugation greatly impacted uptake by HeLa cells (Fig. 4a). Significantly more MHG-FAs (green dots in top left images in Fig. 4a) were found evenly distributed throughout the cytoplasm than MHGs (green dots in left second images from the top in Fig. 4a). Fluorescence quantification using imaging software (Image J) also confirmed significantly more uptake (nearly 5-fold) of MHG-FA than MHG by HeLa cell lines (Fig. 4b). FA conjugation, however, lowered the uptake in other two cell lines tested (MDA in Fig. 4a and b, and Nuff in Figure S4). Negative surface charge of MHG-FA (~−20 mV) presumably lowered the electrostatic interaction of hydrogels with negatively charged cellular membrane than positively charged MHG (~+10 mV).

Co-culture on magnetic field also affected the uptake of hydrogels. Significantly more hydrogels were found in HeLa cells con-cultured on magnet (top right images in Fig. 4a) than non-magnet HeLa cells (top left images in Fig. 4a). Quantification confirmed 3-fold uptake of magnet-guided uptake by HeLa cells (Far left black and gray bars in Fig. 4b). Similar trends were also observed in other cell lines tested. Nearly 2-fold more uptake of hydrogels resulted in magnet co-cultured cells (Fig. 4a,b, and Figure S4). Cells co-cultured on magnet have more chance of interaction with hydrogels as MHGs sediment rapidly due to strong local magnetic field, and hence higher uptake.

Combination of FA conjugation and magnet co-culture resulted in significantly higher uptake by HeLa cells (left second images from the top and top right images in Fig. 4a) which was ~15-fold (Fig. 4b) compared to W/O_FA and W/O_magnet group. This synergistic enhancement was not observed in other cell lines tested due to absence of FR. Folic acid was chosen as the targeting ligand in this study since it is one of the most popular target molecule in cancer drug delivery required for rapid cell growth in many cancer cells^23^. Moreover, our versatile hydrogel preparation method can accommodate conjugation of other targeting molecules such as Arg-Gly-Asp (RGD) peptide or antibodies when desired^30^. Furthermore, secondary targeting achieved by magnetic field is simple and easy readily applicable to subcutaneous tumor models *in vivo*.

### DOX-triggered cytotoxicity and apoptosis

**Figure 5:**
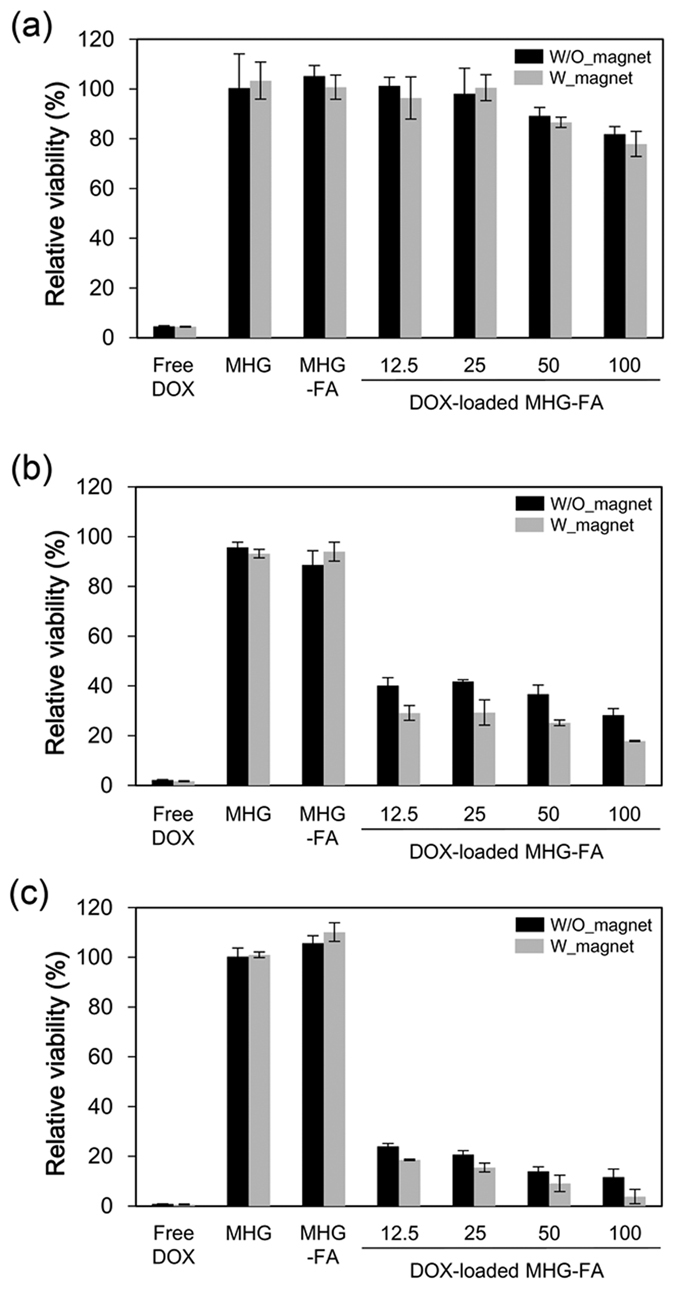
Relative viability of HeLa cells treated with free DOX (100 μg/mL), MHG (100 μg/mL), MHG-FA (100 μg/mL), and DOX-loaded MHG-FAs at various concentrations (12.5, 25, 50 and 100 μg/mL) measured by MTT assay. Cells were treated for 30 min without (W/O_magnet, black bars) or with magnet (W_magnet, gray bars) and further incubated for up to (**a**) 24 h, (**b**) 48 h, and (**c**) 96 h. The relative cell viability was calculated by comparing with absorbance of untreated cells.

DOX is a widely used chemotherapeutic agent known to induce apoptosis through mitotic catastrophe^26^. DOX-loaded MHG-FAs were incubated with HeLa cells at various concentrations (12.5, 25, 50 and 100 μg/mL) along with control groups (free DOX at 100 μg/mL and two DOX-free hydrogels, MHG and MHG-FA at 100 μg/mL). HeLa cells were incubated with each group for limited amount of time (30 min) and washed extensively with PBS to minimize the effect of lately endocytosed hydrogels. After media exchange, cells were further incubated for up to 24, 48 and 96 h (Fig. 5a–c, respectively) and MTT cytotoxicity assay was performed to quantify viable cells. Regardless of incubation time, no-DOX hydrogels (MHG and MHG-FA) showed little to no cytotoxicity (Fig. 5). This shows that our system is a safe delivery method. Magnetite, PNIPAm and FA are all FDA-approved and relatively safe materials and their combination also resulted in a safe drug delivery vehicle^31^,^32^,^33^. FA conjugation slightly increased the cell viability than MHG probably due to decreased zeta potential of MHG-FA by EDC/NHS coupling of primary amines with FA (Table 1).

Free DOX controls showed immediate cytotoxicity exhibiting over 90% cell death as early as 24 h (Fig. 5a far left bars). DOX-loaded MHG-FA (right 4 groups in Fig. 5a–c), on the other hand, exhibited incubation time- and dose-dependent cytotoxicity. As hypothesized, DOX-loaded MHG-FA showed retention in cytotoxic activity due to sustained release of DOX. Increasing the LCST from ~32 °C to ~37 °C by VP copolymerization, achieved sustained DOX release over ~100 h (Fig. 3f) which resulted in slower cytotoxic profile *in vitro*. This can be greatly advantageous especially *in vivo* by avoiding premature DOX release during the circulation and undesired side effects. It is noteworthy that at 96 h, 100 μg/mL DOX-loaded MHG-FA co-cultured on magnet demonstrated significant cytotoxicity almost comparable to that of free DOX control. Magnet response became evident after 48 h incubation. Magnet co-cultured cells (gray bars in Fig. 5) showed significantly higher cytotoxicity than no-magnet cells (black bars in Fig. 5) while free DOX controls showed no response to the magnet existence as expected.

**Figure 6:**
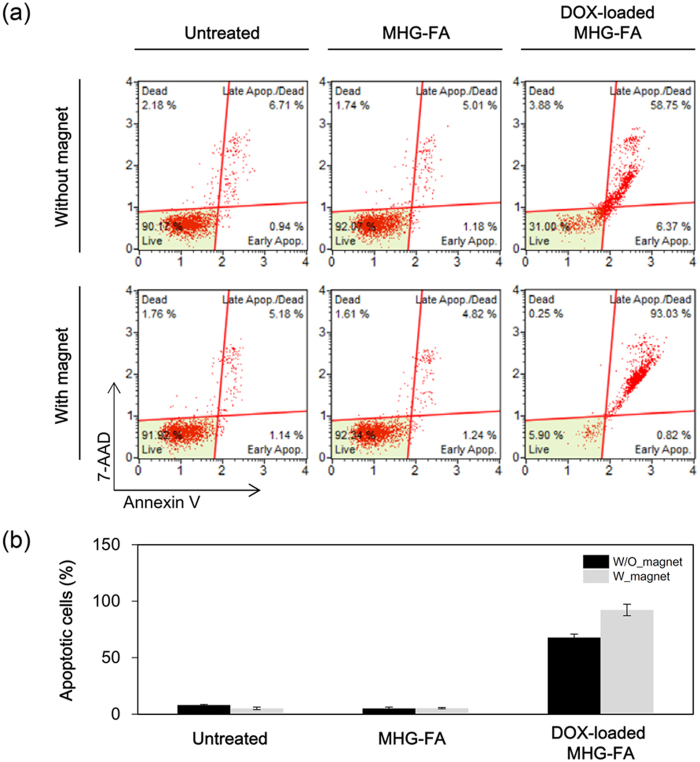
Detection of apoptosis of HeLa cells. Cells were treated with 100 μg/mL MHG-FA or DOX-loaded MHG-FA for 30 min and further incubated for 48 h. Apoptosis was analyzed by Annexin V/7-AAD double staining and flow cytometry. (**a**) Representative results from three independent experiments. The lower right quadrant represents early apoptotic cells stained mainly by Annexin V and the upper right quadrant stands for late apoptotic cells stained by both Annexin V and 7-AAD. (**b**) The percentages of both apoptotic (late and early) and necrotic cells induced by MHG-FA and DOX-loaded MHG FA.

Annexin V and dead cell assay was performed on MHG-FA and DOX-loaded MHG-FA treated cells after 48 h of incubation (Fig. 6a). Results from three independent studies were averaged and shown as a plot in Fig. 6b. MHG-FA incubated cells remained healthy in accordance with MTT assay results. DOX-loaded MHG-FA showed significant increase in apoptotic cells. Negligible amount of necrotic cells confirms that cells are going through DOX-triggered apoptotic stages. Effect of magnet presence greatly impacted HeLa cells showing over 90% of cells in late stage apoptosis.

## Conclusion

In summary, magnetite/hydrogel core-shell hydrogels (MHG) containing magnetic nanoparticles in the core and PNIPAm-based hydrogel shell conjugated with folic acid were prepared as a versatile tool for dual-targeting drug delivery vehicle. Hydrogels were characterized by DLS, SEM, TEM, and UV/Vis. Magnet response and drug release profile were also studied. Influence of the targeting ligand conjugation and the presence of magnet on the intracellular uptake and distribution were observed using CLSM with several cell lines. Finally, efficiency of DOX-loaded MHG-FAs were measured using MTT assay and apoptotic activity assay. In accordance with the authors’ hypothesis, dual-targeting by FA conjugation and magnet-guided incubation synergistically enhanced intracellular uptake of MHG-FAs and therefore increased DOX-triggered apoptosis was realized.

## Methods

### Materials

*N*-isopropylacrylamide (NIPAm) was purchased from TCI (Nihonbashi-honcho, Chuo-ku, Tokyo, Japan). Phosphate buffered saline (PBS) was obtained from Bio Basic Inc. (Markham, Ontario, Canada). Sodium hydroxide (NaOH) and hydrochloric acid (HCl) were purchased from JUNSEI (Nihonbashi-honcho, Chuo-ku, Tokyo, Japan). Fetal bovine serum (FBS), penicillin/streptomycin, Dulbecco’s Modified Eagle Medium (DMEM), and Roswell Park Memorial Institute medium (RPMI) were purchased from Hyclone (Logan, UT, USA). All other chemicals including fluorescein-*o*-methacrylate (FMA), allylamine (AA), and doxorubicin hydrochloride (DOX) were obtained from Sigma-Aldrich (St Louis, MO, USA) and used as received without further purification.

HeLa (human cervical cancer), Nuff (newborn human foreskin fibroblast) and MDA-MB-231 (human breast adenocarcinoma) cells were purchased from the American Type Culture Collection (ATCC) and cultured as recommended. HeLa and Nuff cells were cultured in DMEM supplemented with 10% FBS and 1% penicillin/streptomycin (100 U/ml) at 37 °C in a humidified incubator with 5% CO_2_. MDA-MB-231 cells were cultured in RPMI supplemented with 10% FBS and 1% penicillin/streptomycin (100 U/ml) at 37 °C in a humidified incubator with 5% CO_2_.

### Preparation of FA-conjugated magnetite-hydrogels (MHG-FAs)

Particle core containing MNPs was prepared by precipitation polymerization method under a nitrogen atmosphere. NIPAm (131.7 mg, 1.16 mM), vinyl-2-pyrrolidinone (VP) (18.5 mg, 0.17 mM), *N*,*N’*-methylenbisacrylamide (BisAA) (6 mg, 0.039 mM), FMA (10.0 mg, 0.025 mM) and MNP (0.027 mg, 32 μL) were dissolved in 20 mL of distilled water. MNP was prepared by the co-precipitation method as described in our previous study^34^. The degassed solution was stirred at 250 rpm and heated to 70 °C in an oil bath, and then potassium persulfate (KPS) (4.8 mg, 0.017 mM) was added to initiate the free radical polymerization. The reaction was continued for 3 h under a nitrogen atmosphere. Then, the core particles were dialyzed (MWCO 15,000) against water for 4 days.

Core particles were further shelled by free radical polymerization. A 10 mL of distilled water containing 36 mg of core particles, NIPAm (0.75 g, 0.663 mM), AA (6.8 μL, 0.141 mM) and BisAA (3 mg, 0.019 mM) was degassed for 20 min to remove oxygen, and then KPS (4.8 mg, 0.017 mM) was added. The reaction proceeded for 6 h at 70 °C followed by the dialysis (MWCO 15,000) against water for 4 days. Resultant core-shell hydrogels were designated as magnetite hydrogels (MHGs).

To conjugate FA on the MHGs, *N*-Hydroxysuccinimide (NHS) (1.9 mg, 0.034 mM), 1-Ethyl-3-(3-dimethylaminopropyl)carbodiimide (EDC) (1.3 mg, 0.016 mM) and FA (1.3 mg, 0.006 mM) were first dissolved in 2 mL of dimethyl sulfoxide (DMSO). 17 mg of MHGs were dispersed in 20 mL of PBS solution, then the pH was adjusted to 4.5~4.7 by 1.0 M of HCl. The activated FA solution was added to the MHG solution and stirred at room temperature in the dark for 16 h. The pH was adjusted to 9.0 by 1.0 M NaOH to terminate the reaction and dialyzed (MWCO 15,000) against PBS (pH 7.4) for 3 days and distilled water for 3 days. Resulting MHG-FAs were lyophilized for quantification and storage. All particles were protected from light in order to conserve the fluorescence of FMA for subsequent CLSM experiments.

### Characterization of MHG-Fas

The size and surface charge were measured by DLS (Zetasizer Nano S90, Malvern Instruments, UK) and zeta-potential analysis (Zetasizer Nano ZS90, Malvern Instruments), respectively. The morphology of the lyophilized sample was observed by SEM (JSM-6700F, JEOL, Japan) and TEM (JEM-2010, JEOL, Japan). UV/Vis spectra of samples suspended in 1 mL of distilled water were recorded using a UV-Visible spectrophotometer (V550, JASCO Inc., Easton, MD, USA).

### Drug loading and release

MHG-FAs (3.6 mg) and DOX (0.26, 0.52, and 1.3 mg) were suspended in 3 mL of PBS (pH 7.4) and agitated for 72 h on the shaker at 4 °C in dark. DOX-loaded hydrogels were transferred into a dialysis tube (MWCO 15,000) and then dialyzed against PBS at 37 °C for 24 h. DOX-loaded MHG-FAs were separated from media containing unloaded drug and weighed after freeze-drying. The total amount of the loaded DOX was obtained by measuring the absorbance at 490 nm using a UV-VIS spectrophotometer. Then, the concentration of the DOX was determined with a predetermined calibration curve. The loading efficiency of 27.5, 31.1, and 34.1% was calculated by dividing the total amount of the loaded DOX by the weight of MHG-FAs. Drug release was performed by dialysis at 37 °C in PBS (pH 7.4). The amount of released DOX from MHG-FAs was monitored by measuring the absorbance of diffused DOX in PBS at 490 nm.

### Intracellular distribution and uptake

Intracellular uptake and distribution of hydrogles were investigated by CLSM (LSM-700, Carl Zeiss, Germany). In brief, HeLa, Nuff and MDA-MB-231 cells were seeded in a 12-well culture plate (5 × 10^4^/well) containing 0.1% gelatin-coated 12-mm glass coverslips 24 h prior to inoculation. Then cells were incubated with MHGs and MHG-FAs (at 100 μg/mL) with/without the presence of magnet for 24 h at 37 °C. Cells were then washed with PBS, the cell membranes were stained with Cell Mask^TM^ Orange Plasma Membrane Marker (Invitrogen, Carlsbad, CA, USA) according to the manufacturer’s instructions, fixed with 4% paraformaldehyde (Wako, Osaka, Japan) and the nuclei were stained with Hoechst 33342 (Cell Signaling Technology, Danvers, MA, USA) for 1 h according to the manufacturer’s protocol and washed again with PBS. The cells were scanned in three dimensions as a z-stack of two-dimensional images and an image cutting horizontally through approximately the middle of the cellular height was selected to differentiate intracellular fluorescence from those on the cellular surface. Each emission light was scanned separately for individual excitations of the dyes to eliminate fluorescence cross-talk. Numbers of green pixels were counted using Image J software in order to quantify relative uptake of hydrogels.

### DOX-responsive cell viability and apoptosis assay

Cytotoxicity of the MHG, MHG-FA and DOX-loaded MHG-FA was determined by MTT assay. HeLa cells were plated into 96-well plates at a density of 2 × 10^4^ cells/well 24 h prior to treatment. After cell attachment, media were replaced with complement media containing various hydrogels at 37 °C in a humidified 5% CO_2_ incubator. After 24 h, 48 h and 96 h, 10 μL of MTT solution (stock prepared at 5 mg MTT in 5 ml media) replaced the media and cells were further incubated for 2 h at 37 °C. Formazan precipitate was dissolved in DMSO, and the absorbance at 550 nm was measured using a microplate reader (FLUOstar OPTIMA, BMG LABTECH, Cary, NC, USA). The relative cell viability (%) was calculated as (OD of treated cells/OD of non-treated cells) × 100. Apoptotic activity of cells was measured using MUSE^TM^ Annexin V and Dead Cell kit (Millipore, Billerica, MA, USA), following the manufacturer’s protocol. Briefly, after incubation with DOX-loaded hydrogels for 24, 48, 72 and 96 h, cell pellets from each group were collected by centrifugation (1,200 rpm, 3 min) and were resuspended in complete media. And the Muse Annexin V and Dead Cell Dye assay kit reagent was added (100 μl to each tube). After incubation for 20 min at room temperature in the dark, cells were analyzed using a Muse Cell Analyzer (Merck Millipore).

### Statistics

Data from repeated experimentations is shown as mean ± standard error of the mean.

## Additional Information

**How to cite this article**: Kim, H. *et al*. Synergistically enhanced selective intracellular uptake of anticancer drug carrier comprising folic acid-conjugated hydrogels containing magnetite nanoparticles. *Sci. Rep.* **7**, 41090; doi: 10.1038/srep41090 (2017).

**Publisher's note:** Springer Nature remains neutral with regard to jurisdictional claims in published maps and institutional affiliations.

## Supplementary Material

Supplementary Information

## Figures and Tables

**Table 1:** Size, PDI and surface charge of hydrogels.

	Diameter [nm]	PDI	Zeta potential [mV]
Core	160.2	0.111	−9.0
MHG	191.2	0.157	+10.2
MHG-FA	219.5	0.125	−20.6
