# Supplementary material for: Synergistically enhanced selective intracellular uptake of anticancer drug carrier comprising folic acid-conjugated hydrogels containing magnetite nanoparticles
Source: Sci Rep. 2017 Jan 20;7:41090. doi: 10.1038/srep41090 (PMC5247690; doi:10.1038/srep41090)
Supplement: Supplementary Information [file srep41090-s1.pdf]

<Supplementary Information>

Synergistically enhanced selective intracellular uptake of anticancer drug carrier comprising folic acid-conjugated hydrogels containing magnetite nanoparticles

Haneul Kim[a,+], Ara Jo[b,+], Seulgi Baek[a,+], Daeun Lim[a], Soon-Yong Park[b], Soo Kyung Cho[a,], Jin Woong Chung[b,]*, and Jinhwan Yoon[a,]**

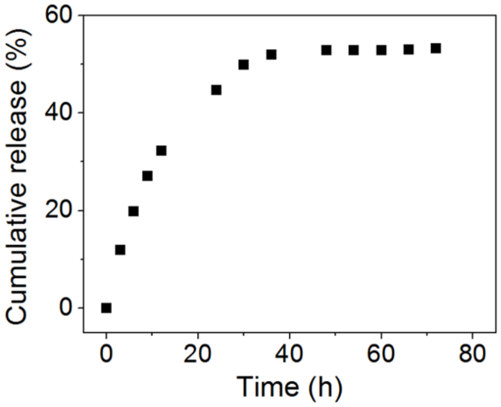

Figure S1. DOX release from pure PNIPAm hydrogels over time at 37 °C.

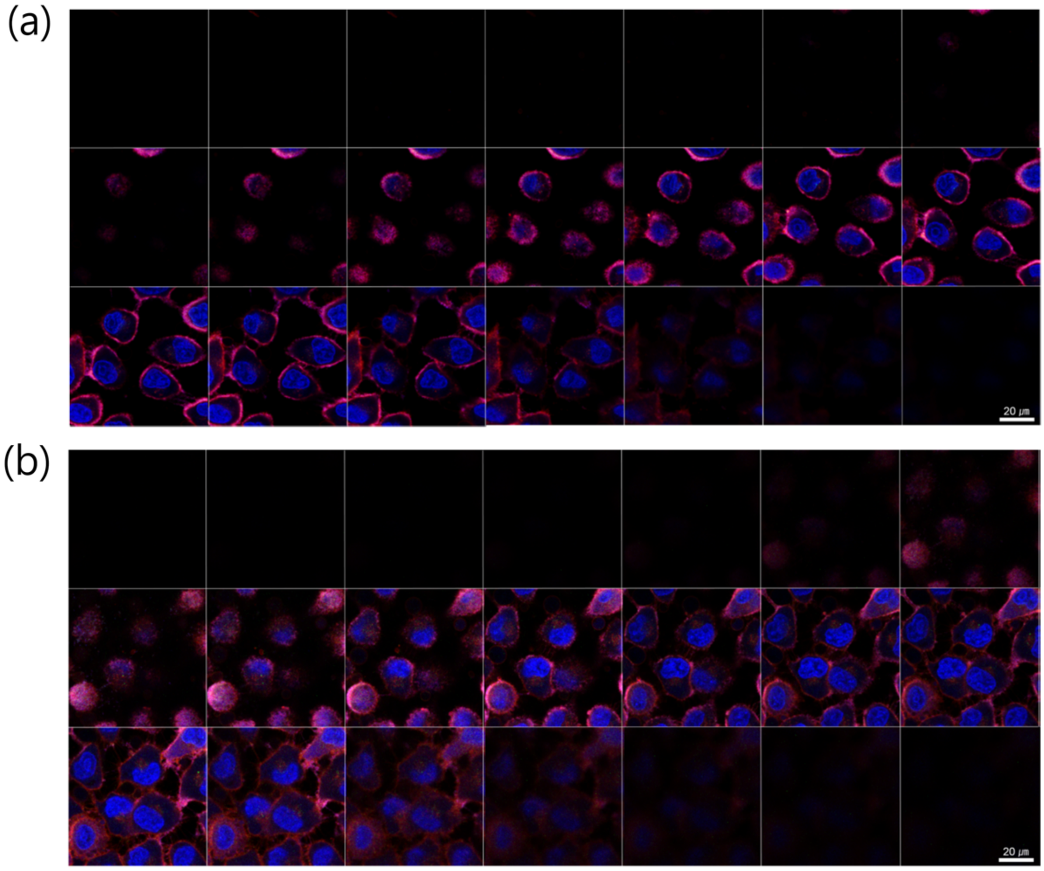

Figure S2. Z-stack images of HeLa cells recorded using CLSM. Cells were incubated with 100 μg/mL of (a) MHG-FA (W_FA) without magnet (top left images in Figure 4a) and (b) MHG-FA (W_FA) with magnet (top right images in Figure 4a) for 24 h at 37 ^{o}C. Nuclei were labeled blue with Hoechst 33342 and cell plasma membranes were stained orange.

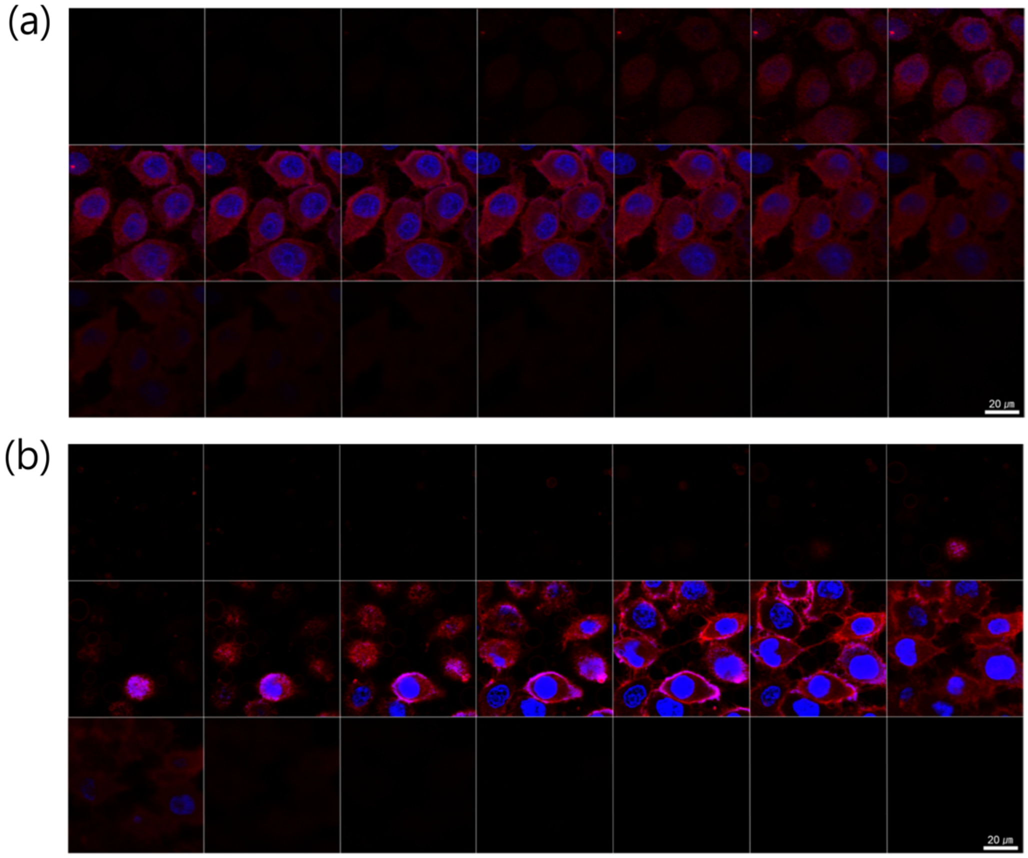

Figure S3. Z-stack images of HeLa cells recorded using CLSM. Cells were incubated with 100 μg/mL of (a) MHG (W/O_FA) without magnet (second top left images in Figure 4a) and (b) MHG (W/O_FA) with magnet (second top right images in Figure 4a) for 24 h at 37 ^{o}C. Nuclei were labeled blue with Hoechst 33342 and cell plasma membranes were stained orange.

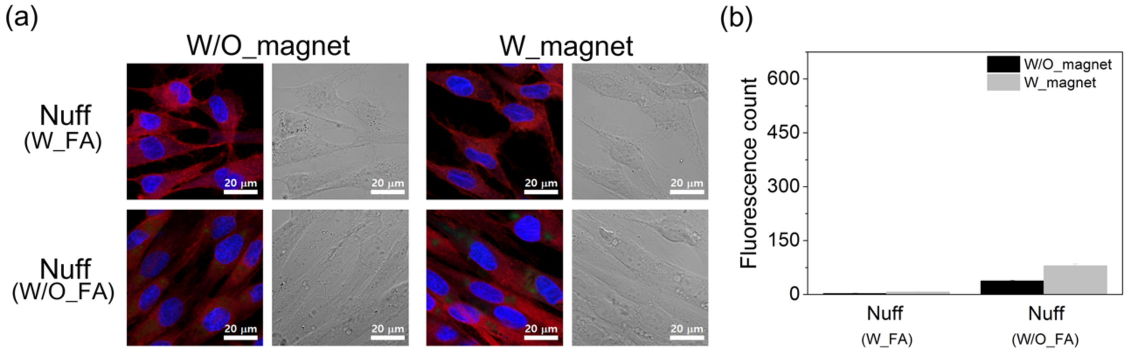

Figure S4. Intracellular localization and cellular uptake of hydrogels by Nuff. (a) Intracellular localization of hydrogels in Nuff cell recorded using CLSM. Cells were incubated with 100 μg/mL of MHG-FA (W_FA) or MHG (W/O_FA) without magnet (W/O_magnet, images on the left) or with magnet (W_magnet, images on the right). Nuclei were labeled blue with Hoechst 33342 and cell plasma membranes were stained orange. (b) Quantitative cellular uptake of hydrogels by Nuff cell counted using Image J.
